# Giant colonic lipoma with prolapse through the rectum treated by external local excision: A case report

**DOI:** 10.3892/ol.2014.2317

**Published:** 2014-07-04

**Authors:** EMIN KOSE, GOKHAN CIPE, SERDAR DEMIRGAN, SUKRU OGUZ

**Affiliations:** 1 Department of General Surgery, Büyükcekmece State Hospital, Istanbul 34500, Turkey; 2 Department of General Surgery, Bezmialem Vakif University School of Medicine, Istanbul 34093, Turkey; 3 Department of Anesthesiology and Reanimation, Bagcilar Education and Research Hospital, Istanbul 34200, Turkey; 4 Department of Radiology, KTU Farabi Hospital, Trabzon 61080, Turkey

**Keywords:** colonic lipoma, colonoscopy, external excision

## Abstract

Colonic lipomas are a rare type of gastrointestinal benign tumor. Those that are <2 cm are generally asymptomatic and do not require any treatment. However, those that are >2 cm may be symptomatic, resulting in abdominal pain, changes in bowel habits, intestinal obstruction and rectal bleeding. A 39-year-old male patient presented with a mass prolapse through the anal canal, which was causing anal pain and rectal bleeding. The patient was admitted to hospital via the emergency services and directed to the Department of General Surgery with the preliminary diagnosis of a rectal prolapse. A pedunculated polyp (size, 10×8×7.5 cm) was detected at the 35th cm of the anal canal. Due to the large size of the polyp, an endoscopic polypectomy could not be performed. Therefore, the prolapsed lipoma was excised externally and the patient was discharged on the first postoperative day on which no complications were experienced. A colonic lipoma must be considered during the differential diagnosis of anorectal diseases, such as hemorrhoids and rectal prolapses. Local excision, hemicolectomy, and segmental and external resection should be considered in addition to an endoscopic polypectomy for the diagnosis and treatment of colonic lipomas.

## Introduction

Colonic lipomas are an uncommon type of benign gastrointestinal tumor. Although colonic lipomas are generally asymptomatic, when the colonic lipoma measures >2 cm, certain symptoms, such as abdominal pain, change in bowel habits, bleeding and a mass that presents as a rectal prolapse may be observed (1,2). While colonic lipomas are often observed in the caecum and ascending colon, they may also be observed in the transverse, descending, and sigmoid colon and in the rectum (3). In the current study, the case of a patient exhibiting a giant, rarely observed lipoma of the sigmoid colon is presented with a description of its external excision. Patient provided written informed consent.

## Case report

**Figure 1:**
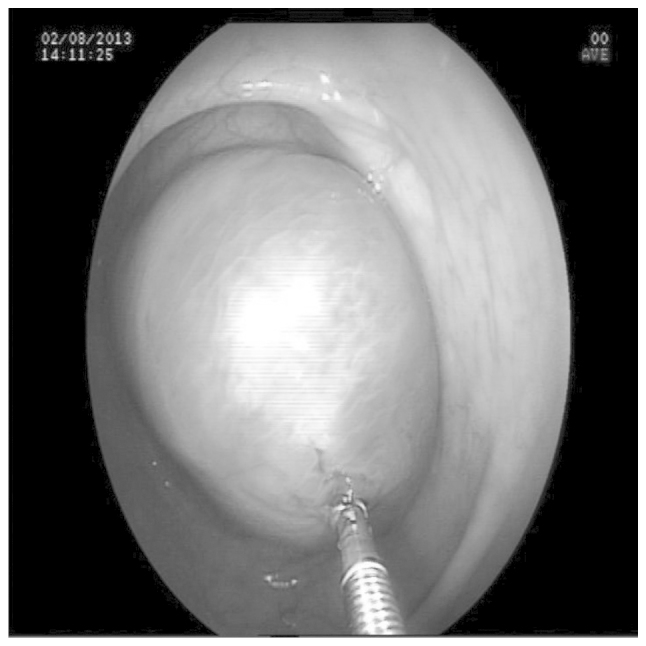
Colonoscopic image of the lipoma.

**Figure 2:**
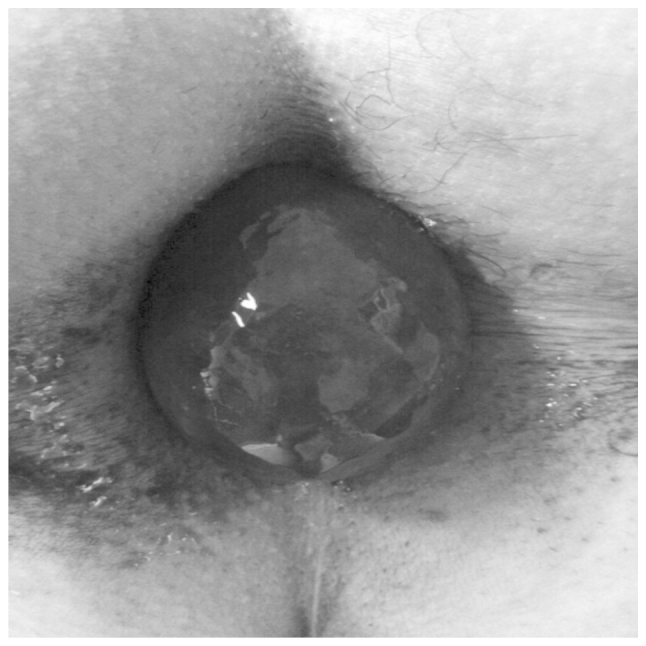
Mass protruding from the anal canal.

**Figure 3:**
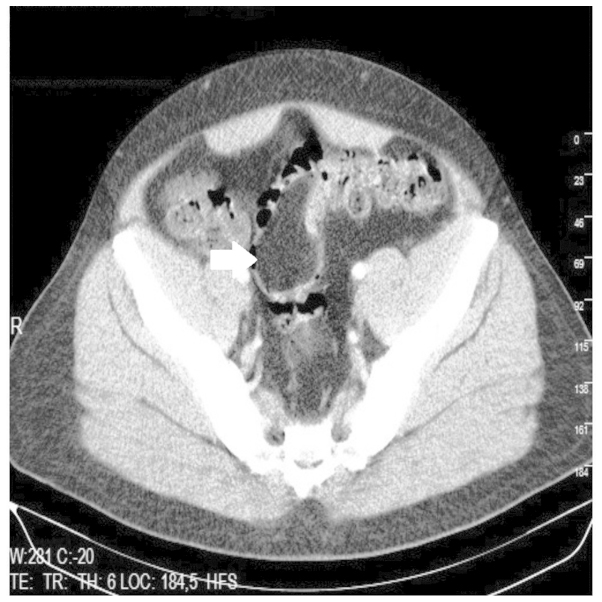
Computed tomography image of the mass (arrow).

**Figure 4:**
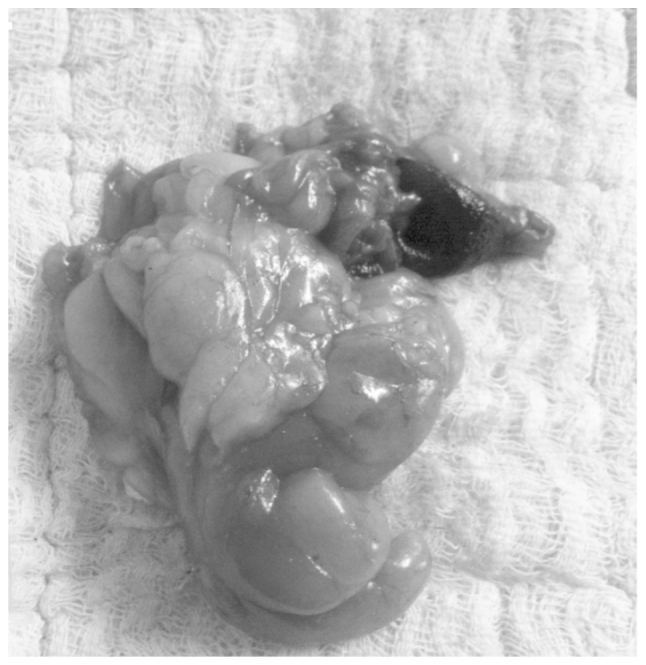
Gross lipoma specimen.

A 39-year-old male patient was admitted to our emergency clinic with a mass protruding from the anal canal. Other symptoms included anal pain and rectal bleeding. During the physical examination, the prolapsed mass was spontaneously reduced through the rectum. The mass was initially diagnosed as a rectal prolapse and the patient was transferred to the Department of General Surgery, Buyukcekmece State Hospital (Istanbul, Turkey) in June 2012. During the physical examination, the mass was forced out from the anal canal while the patient performed the Valsalva maneuver under light sedation. The mass (size, 10×8 cm) had a shiny surface and hyperemia was detected during the physical inspection (Fig. 1). A smooth-surfaced soft tissue with a pedicle (diameter, 3 cm) was detected on palpation. The mass was reduced manually by gentally pushing it through the anus. The routine laboratory examination results were normal and no abnormalities were noted in the patient’s background or family history. A colonoscopy was performed, and at the 35th cm of the anal canal, a mobile, shiny, hyperemic, smooth-surfaced, giant polyp (size, 10×8×7.5 cm), which was covered by a mucosa and a pedicle (diameter, 3 cm) was occluding almost all of the lumen (Fig. 2). At the sigmoid colon, an 8×6-cm fat-density lesion (representing the lipoma) was observed via abdominal computed tomography (CT; Fig. 3). An endoscopic polypectomy was attempted, however, it was unsuccessful due to the size of the lesion measuring ~10 cm in its maximum diameter making it difficult to manipulate the lesion. As a result, an external excision was scheduled. Under light sedation the patient performed the Valsalva maneuver and the mass was forced out from the anal canal. In order to perform an easy removal of the mass from the anus, the pedicle at the exit of the anal canal was tied and the patient’s depth of anesthesia was strengthened. The mass was pulled out, and the distal end of the pedicle was tied and excised. The resected specimen underwent histopathological examination (Fig. 4), which revealed the mass to be a submucosal lipoma with a normal mucosa. The patient was discharged on the first postoperative day on which he did not experience any complications. The colonoscopy, which was performed two weeks later, was considered to be normal.

## Discussion

Colonic lipomas are rarely observed, and are an asymptomatic, benign, submucosal and nonepithelial type of gastrointestinal tumor. In general, this type of lipoma is <2 cm, sessile or pedunculated and often located in the right colon. These lesions are more common in women, observed in the fifth and sixth decades of life and are usually detected during an autopsy, colonoscopy or surgery (2). Lipomas that are >2 cm may be symptomatic, however, in cases where the lipoma is large, it may result in abdominal pain, change in bowel habits, intestinal obstruction and rectal bleeding (4,5). The current case is considered to be rare due to the location of the lesion, its size and the symptom of external prolapse.

Colonic lipomas are generally submucosal lesions with a smooth mucosal surface, which are composed of lobular adipose tissues (2). The colonoscopic properties are distinguished by the lesion’s soft and smooth surface, which is covered by a yellow-colored mucosa (6). Typical characteristics of this type of lipoma include tenting of the tissue (when the mucosa covering the lesion is removed using forceps), collapse of the lesion when pressure is applied to it, in addition to yellow-colored adipose tissue, which may be observed during biopsy (1,2,7). The colonoscopy in the present study revealed a giant polyp that was mobile, shiny, hyperemic, smooth-surfaced, covered by a mucosa and exhibited the abovementioned characteristics. The mass measured 10×8×7.5 cm and the pedicle diameter was 3 cm, which occluded almost all of the lumen. Endoscopic biopsies for colonic lipomas provide only a limited diagnosis, and the pathology results are generally normal or demonstrate an ulcerated mucosa (2). A biopsy was not necessary in the present case as the mass was externally prolapsed.

Imaging techniques facilitate diagnosis; however, as the results are not definitive, the final diagnosis may only be obtained following excision of the tissue (1,2,6,8). A smooth oval filling defect may be observed via barium X-rays, while non-vascular, hyperechoic, submucosal lesions that are observed using endoscopic ultrasonography may supplement the diagnosis, however, are not specific to the diagnosis (8,9). For large colonic lipomas (>2 cm), CT is able to detect masses by demonstrating sharp margins in the homogeneous fat density and magnetic resonance imaging distinguishes lesions in the adipose tissue intensity (6,8). In the present case, CT was adopted as the imaging method and an 8×6-cm fat-density lesion (representing the lipoma) was observed in the sigmoid colon.

Endoscopic polypectomy, local excision, hemicolectomy, or segmental resections are performed for the treatment of colonic lipomas, depending on the popularity and reliability of the particular technique. Endoscopic polypectomy is the preferred treatment strategy, particularly for small lipomas (<2 cm). Although, the risk of bleeding and perforation is high when performing an endoscopic polypectomy on polyps >2 cm (4,9). Kim *et al* (4) demonstrated that it was possible to excise lipomas <3.8 cm, following a submucosal saline injection, without the patient experiencing any complications. Jiang *et al* (2) proposed that surgical procedures are required in the following instances: i) when the lipoma is sessile or has a pedicle >4 cm; ii) the lipoma is malignant; iii) the patient exhibits symptoms, such as intussusception; iv) a muscular layer or serosal attachment is present; or v) the lesion cannot be removed by colonoscopy. There are various surgical procedures that may be conducted for lipomas, including hemicolectomy, segmental resection or local excision (8). In the present case, since the mass occurred as an external prolapse and had a pedicle, external resection was performed to obtain the final diagnosis, thus, no major surgical procedure was required.

In conclusion, colonic lipoma should be considered in the differential diagnosis of anorectal diseases, such as hemorrhoids and rectal prolapse. An external excision may be performed for the treatment of a prolapsed colonic lipoma, as this procedure is considered to be safe and reliable. Furthermore, it may be performed as an alternative to major surgery in certain patients.
